# Society Meetings

**Published:** 1909-10

**Authors:** 


### SOCIETY MEETINGS.

---

The American Association of Obstetricians and Gynecologists held its Twenty-Second Annual Meeting at Fort Wayne, September 21-23, 1909, under the presidency of Dr. William H. Humiston, of Cleveland. It was a meeting full of interest, one of the best in the history of the association. The attendance was large and the enthusiasm was great. The next annual meeting will be held at Syracuse, under the presidency of Dr. A. B. Miller, of that city.

---

The Forty-Seventh University Convocation of the State of New York, will be held in the Senate Chamber at Albany, Thursday evening, Friday and Saturday, October 28, 29, 30, 1909. Sessions begin promptly at 9.30 A. M., 3 P. M., and 8 P. M.

The program contains many interesting numbers and the convocation promises to be a profitable one.

---

The Eighth District Branch of the Medical Society of the State of New York, held its Fourth Annual Meeting at Buffalo, September 8-9, 1909, under the presidency of Dr. E. E. Snow, of Batavia.

An interesting program was disposed of and, in the evening of the first day, a dinner was served at the University Club, which was well attended. Several good speeches were made, notably those by Reverend George B. Richards, rector of the Church of the Ascension, of Buffalo, Judge Safford E. North of Batavia, and Dr. Charles G. Stockton, president of the Medical Society of the State of New York.

Mr. Richards among other things said that the various manifestations of psychotherapy were today the strongest possible evidence of the lack of coöperation between the church and the medical profession.

"And the pity of it all is," said Mr. Richards, "that you and I have slipped a cog and let this thing come in. Somehow we seem to have overlooked the fact that in dealing with men we have to deal with body, mind and soul. I know not what has been the cause of this lack of sympathy, whether it has been the narrow dogmatism of the church or the intellectual pride of the physicians; however you account for it, the fact remains there has been a coldness between the church and science, between the physician and the clergyman."

The speaker proceeded to criticise the doctors who are fond of saying they can't believe in miracles. Some physicians seemed to think because they were specialists in science, they were able to solve the problems of theology. This was an untenable position, he said, for the average practitioner who prated about the incredibility of this or that in the Bible, had but the most superficial knowledge of the subject.

But Mr. Richards did not stop there. What he gave to the doctors he passed out to the members of his own profession, when he criticised ministers for dabbling in mental healing, which, according to his present thinking, was quite outside the province of the church. There was a time when the church controlled art, music, science and philanthropy, but that time has past. Today the church inspires the best in all these branches of human endeavor without seeking to control them.

"Leave psychotherapy where it belongs," said he, "in the hands of the medical profession. I do not think the average clergyman knows enough about psychology to practise it with safety or with profit to those he seeks to benefit."

These were elected officers for the ensuing year: president, E. Munson of Medina; vice-presidents, T. H. McKee of Buffalo and J. S. Wright of Perry; secretary, C. S. Tompkins, Buffalo, and treasurer, C. A. Wall, Buffalo.

---

The Thirty-Fifth Annual Meeting of the Mississippi Valley Medical Association, will be held at St. Louis, Mo., October 12, 13, 14, 1909. Headquarters, general sessions, medical and surgical sections and exhibits are to be located at the Southern Hotel.

The program is large and the papers promise interest. The officers are: J. A. Witherspoon, president, Nashville; Louis

Frank, first vice-president, Louisville; Albert E. Sterne, second vice-president, Indianapolis; Henry Enos Tuley, secretary, Louisville; Samuel Cecil Stanton, treasurer, Chicago; Louis H. Behrens, chairman of the Committee of Arrangements.

